# Unveiling the Financial Burden of Systemic Lupus Erythematosus Management in Saudi Arabia: Insights from a Single-Center Study

**DOI:** 10.3390/healthcare13233075

**Published:** 2025-11-26

**Authors:** Aseel Alsuwayegh, Yazed AlRuthia

**Affiliations:** 1 Corporate Department of Pharmacy Services, King Saud University Medical City, Riyadh 11451, Saudi Arabia; aalsuwayegh@ksu.edu.sa; 2 Department of Clinical Pharmacy, College of Pharmacy, King Saud University, Riyadh 11451, Saudi Arabia

**Keywords:** systemic lupus erythematosus, healthcare cost, Saudi Arabia, lupus nephritis, biologics

## Abstract

Background: Systemic lupus erythematosus (SLE) is a chronic autoimmune disorder that impacts multiple organs. In Saudi Arabia, the prevalence of SLE is about 19 per 100,000 people, primarily affecting women at a 9:1 ratio. This study aimed to estimate the direct medical costs of managing SLE in Saudi Arabia. Methods: Data were collected from electronic medical records at a university-affiliated tertiary care center using a micro-costing approach over a 12-month period, encompassing the costs for laboratory tests, imaging, medications, and outpatient visits. A Generalized Linear Model (GLM) was used to assess the relationship between costs and patient characteristics. Results: A total of 103 patients were observed, who were primarily female (90.23%), Saudi nationals (97.09%), and married (53.40%). The average age was 41.27 years (±12.01), with an illness duration of 13.37 years (±6.64). Approximately 44% had lupus nephritis, 13% had thrombocytopenia, 20% had leukopenia, 9% had neuropsychiatric lupus, 44% had arthritis, and 20% had chronic cutaneous lupus. Approximately 60% exhibited mild or no disease activity, as per the SLEDAI-2K score. The mean annual medical cost associated with the management of systemic lupus erythematosus (SLE) was USD 12,760.65. However, after adjusting for various covariates using the Generalized Linear Model (GLM), the estimated annual medical costs were USD 23,041 for patients treated with biologics and USD 47,793 for those with high disease activity. Both high disease activity (SLEDAI-2K score > 11) and the use of biologics were identified as significant predictors of increased annual medical costs. Conclusions: This study is the first to estimate the costs of SLE management in Saudi Arabia from a public healthcare perspective. Future research should include a larger, more representative sample and consider the productivity losses associated with SLE.

## 1. Background

Systemic lupus erythematosus (SLE) is a chronic autoimmune disorder that poses significant public health challenges globally, particularly impacting non-Caucasian populations [1]. A systematic analysis estimates the global incidence of SLE at 5.14 cases per 100,000 person-years and a prevalence of 43.7 per 100,000 individuals [2]. In the Arabian Gulf, including the UAE, SLE presents distinct challenges; a study conducted from 2009 to 2012 identified 16 new cases among native Arabs, yielding an incidence rate of 8.6 per 100,000 and a prevalence rate of 103 per 100,000, with a notable female predominance [3,4]. In Saudi Arabia, the prevalence rates vary significantly: a 2002 study in Al-Qassim reported 19.28 per 100,000, whereas a 2015 study in Taif reported an exceptionally low prevalence of 0.038% [5,6]. Additional research underscored that the overwhelming majority of patients affected by SLE are female, aligning with global demographic trends [7,8].

The clinical presentation of systemic lupus erythematosus (SLE) in the Arab world is characterized by a diverse range of features, revealing both commonalities and unique aspects when compared to European and Latin American populations [9,10]. A particularly notable complication in the Arabian Gulf is lupus nephritis (LN), with a systematic review of studies from this region indicating that LN accounts for approximately 10% to 36% of all renal biopsies and affects nearly half (around 48%) of SLE patients in a large cohort from Saudi Arabia [3,11]. Supporting this, a single-center study in the Kingdom of Saudi Arabia found that LN was present in approximately 40% of patients [7].

The most frequently reported clinical feature among Arab patients, according to pooled analyses, is arthralgia/arthritis (approximately 81.1%), followed by anemia (55.6%), fatigue (53.4%), malar rash (53.1%), and renal involvement (50.4%) [10]. More recent studies from single centers in Saudi Arabia have reinforced these observations, highlighting joint involvement as the most affected organ (76.2%) in one cohort, along with prevalent symptoms such as joint pain (91%), hair loss (82%) [7], and skin rash (61%) in another study [8].

Laboratory findings consistently show that nearly all patients are positive for antinuclear antibodies (ANA) (ranging from 95.2% to 100%) and demonstrate a high prevalence of anti-double-stranded DNA (anti-dsDNA) antibodies (ranging from 68.6% to 75%) [4,7,10]. A systematic review conducted by Alenzi et al. in 2025 focusing on extrarenal manifestations in Saudi patients underscored substantial variability in findings across studies [9]. This variability was attributed to differences in study design and a lack of standardized definitions. Notable findings on the prevalence of extrarenal features in Saudi Arabia populations included musculoskeletal manifestations (2% to 100%), mucocutaneous involvement (5% to 100%), constitutional symptoms (e.g., fatigue, which ranges from 40% to 64.8%), neuropsychiatric features (approximately 1.5% to 67.6%), and hematologic manifestations (e.g., anemia, which ranges from 5% to 89%) [9]. The review highlighted that certain features, such as renal involvement and specific autoantibodies, were more frequently observed in Arab populations than in published cohorts from Europe and Latin America [9].

Treatment in the Arabian Gulf region generally follows international guidelines such as those of the Asia Pacific League of Associations for Rheumatology (APLAR), European Alliance of Associations for Rheumatology (EULAR), and Kidney Disease: Improving Global Outcomes (KDIGO) [3]. Hydroxychloroquine (HCQ) is integral to management, as demonstrated by its usage in a Saudi cohort, where it was reported to be used by 99% of patients, followed by steroids, which were utilized by 84.8% of participants [7]. For the induction therapy of lupus nephritis (LN), cyclophosphamide and mycophenolate mofetil (MMF) are commonly employed, with MMF often preferred for maintenance therapy alongside azathioprine and corticosteroids [3]. A 2023 review by Pawlak-Buś et al. emphasized a global shift towards a ‘treat-to-target’ approach, which aims to achieve remission or low disease activity while minimizing glucocorticoid exposure [12]. This review further highlighted HCQ as a cornerstone of long-term management. It discusses the growing importance of biologic therapies, such as belimumab and anifrolumab, as well as new small-molecule inhibitors [12].

Outcomes for patients with SLE in the region vary considerably. In a study conducted in the UAE, remission rates for lupus nephritis (both complete and partial) were 56% and 37% at 6 months, respectively [3]. However, a subset of patients progresses to end-stage renal disease (ESRD), with rates documented to be between approximately 6% and 9% in a systematic review [3]. Survival outcomes are generally promising, with reported five- and ten-year survival rates of 92% and 77%, respectively [3]. The pooled mortality rate among Arab patients stands at approximately 7.6% [10]. In terms of Quality of Life (QoL), a cross-sectional study in Saudi Arabia found an average Health-Related QoL (HRQoL) score of 57.09 ± 18.81, with the lowest scores observed in the emotional health domain (mean: 44.67 ± 30.00) [8]. Notably, the presence of comorbidities such as cardiovascular disease, diabetes, and hypertension emerged as the sole significant determinant of lower QoL [8,10].

SLE imposes a significant economic burden in the region. A five-year economic impact study in the UAE estimated the total cost of SLE from both payer and societal perspectives at AED 3 billion and AED 10 billion, respectively [13]. The most significant portion of expenses, amounting to AED 7 billion, was attributed to productivity loss, highlighting the substantial societal cost associated with the disease [13]. The estimated cost per patient per year from a societal perspective was AED 148,468 (approximately USD 66,578) [13]. Delayed diagnosis of SLE poses a major challenge in Saudi Arabia. A multicenter cross-sectional study revealed that 82.3% of patients experienced at least one delay in diagnosis [14]. The most significant bottleneck identified was delayed rheumatology referral, affecting 65.7% of patients. Additionally, the specialty of the first physician consulted (particularly in primary care and dermatology) and the number of physicians seen before receiving a diagnosis were significantly associated with these delays [14]. These delays could contribute to faster disease progression and increased disease burden. Despite the higher observed incidence rates of SLE in Saudi Arabia over the past decade [14], no study has estimated the direct medical costs of SLE management in the Kingdom. Therefore, this study aimed to estimate the direct medical costs of SLE management in Saudi Arabia and identify the key drivers of these costs.

## 2. Materials and Methods

### 2.1. Study Design and Setting

This investigation used a retrospective, single-center cohort study design to assess the direct medical costs associated with SLE in Saudi Arabia. The research was conducted at King Khalid University Hospital, a major tertiary care educational institution in Riyadh, which provided the data for this study. A thorough extraction process was undertaken utilizing the hospital’s electronic health records. The defined retrospective data collection period extended from 21 March 2020 to 30 December 2021. This timeframe enabled an in-depth examination of patient records, treatment regimens, and associated medical costs, providing valuable insights into the economic impact of SLE on both patients and the healthcare system in Saudi Arabia.

### 2.2. Patient Selection Criteria

The study included adult patients aged 18 years or older with SLE who received treatment according to standard care protocols. These treatments included corticosteroids and hydroxychloroquine, as well as biologics such as belimumab, rituximab, and anifrolumab, in combination with standard therapy. To ensure thorough follow-up for the cost analysis, only patients who had completed at least 12 months of treatment were included, all of whom were 20 years of age or older. Patients under 18 years had only 1 or 2 visits, making it challenging to gather sufficient data to accurately estimate the cost of SLE management for this age group.

The exclusion criteria were individuals under 18 years of age (pediatric patients) and those with specific comorbidities that could significantly impact cost attribution. These comorbidities included cancer (both hematologic and solid tumors), chronic kidney disease, cardiovascular disease, and stroke.

### 2.3. Data Collection

Data was extracted from electronic medical records (EMRs) and included a range of demographic, clinical, and resource utilization variables:

- Demographics and History: Age, gender, duration of SLE illness (in years), and presence of comorbid medical conditions (e.g., diabetes, dyslipidemia, and hypertension).
- Healthcare Utilization and Costs: Information necessary for the cost estimation, such as prescription medications dispensed, number of hospital admissions and corresponding length of stay (days), types of laboratory tests performed, imaging studies conducted, and frequency of outpatient visits and Emergency Department (ER) visits.
- Disease Activity: SLE disease activity was measured using the Systemic Lupus Erythematosus Disease Activity Index 2000 (SLEDAI-2K) [15,16]. The SLEDAI-2K is a widely accepted clinical tool that quantifies lupus disease activity over the preceding 10 days [16]. This index incorporates 24 weighted clinical and laboratory manifestations and requires physician assessment and lab results for scoring [15]. Disease activity was stratified based on the SLEDAI-2K scores as follows [16]:
  ○ No activity: SLEDAI-2K = 0;
  ○ Mild activity: SLEDAI-2K = 1−5;
  ○ Moderate activity: SLEDAI-2K = 6−10;
  ○ High activity: SLEDAI-2K = 11−19;
  ○ Very high activity: SLEDAI-2K ≥ 20.

### 2.4. Costing Methodology and Perspective

This direct medical cost estimation project was conducted from the perspective of public healthcare payers in Saudi Arabia. All healthcare services utilized by the SLE cohort at the tertiary center were documented in the EMRs. This documentation included a comprehensive range of resource categories, such as outpatient consultations, laboratory tests, imaging studies, dispensed medications, emergency room visits, and hospitalizations.

To estimate costs, a micro-costing approach was used, enabling the assignment of precise monetary values to each service rendered. The prices for healthcare services were obtained from the officially published rates from the Saudi Council of Health Insurance (CCHI) [17]. Additionally, the costs of prescription medications were sourced directly from the Saudi Food and Drug Authority (SFDA) website and discounted based on the inputs of the pharmacy and planning specialists since public hospitals procure their medication and medical device needs through centralized tenders [18].

### 2.5. Statistical Analyses

To ensure patient confidentiality, all collected data were anonymized. Data extraction and initial management were conducted using Microsoft Excel (version 2019, Microsoft, Redmond, WA, USA). Descriptive statistics were used to summarize the baseline demographic and clinical characteristics of the SLE patient cohort, as well as the direct medical costs incurred. These summaries included frequencies and percentages for categorical variables, along with means and their corresponding 95% confidence intervals (CIs) for continuous variables such as cost. The overall unadjusted total annual direct costs were presented as the mean, 95% CIs for the mean, and the median, along with the lower and upper quartiles. To identify predictors of healthcare expenditure and estimate the adjusted mean annual direct medical cost per SLE patient, we applied a Generalized Linear Model (GLM). A gamma distribution with a log-link function was chosen for the GLM analysis, which is often regarded as an optimal choice for GLMs in healthcare expenditure analysis, primarily because it effectively addresses two significant statistical challenges: skewness and heteroskedasticity. Its inherent flexibility makes it particularly well-suited to handle the pronounced right skewness commonly found in cost data, often outperforming models that use log transformations and ordinary least squares (OLS) [19]. The model included factors such as age, duration of illness, use of biologic therapies, gender, and disease activity, which were assessed using the SLEDAI-2K scale, as follows:

g(E[Overall Healthcare Costs]) = β_o_ + β_1_ Age + β_2_ Duration of Illness + β_3_ Gender + β_4_ Disease Activity + β_5_ Use of Biologics

Multicollinearity was evaluated using the variance inflation factor (VIF). None of the calculated VIF values exceeded 1.2, indicating no significant collinearity. All cost figures were converted from Saudi Riyals (SAR) to US Dollars (USD) using a fixed exchange rate of 3.75 SAR to 1.00 USD. Statistical analyses were conducted using SAS^®^ software version 9.4 (SAS^®^ Institute, Cary, NC, USA).

### 2.6. Ethical Considerations

The research protocol was granted ethical approval by the Research Ethics Committee at the College of Medicine, King Saud University (IRB Approval of Research Project No. E-21-5775). To uphold patient privacy and confidentiality, the dataset was de-identified entirely, ensuring that no personal identifiers, such as names, medical record numbers, addresses, or national ID numbers, were included. The study was conducted in strict accordance with the principles of research ethics as defined in the Declaration of Helsinki [20].

## 3. Results

### 3.1. Baseline Characteristics of the Study Cohort

The analysis included 103 adult patients diagnosed with SLE. The cohort was predominantly female (90.29%, n = 93) and primarily consisted of Saudi nationals (97.09%, n = 100) (Table 1).

The age distribution indicated that the majority of patients were between 20 and 59 years old, with the 40–59 age group accounting for the largest segment (49.51%, n = 51). The duration of the disease was notably prolonged, with 68.93% (n = 71) of patients having lived with SLE for a decade or longer.

In terms of clinical manifestations and disease complications, arthritis and lupus nephritis were the most common, each affecting 43.69% (n = 45) of the cohort. Other prevalent complications included alopecia (22.33%, n = 23), proteinuria (20.39%, n = 21), and leukopenia (20.39%, n = 21).

The distribution of baseline disease activity, as measured by the SLEDAI-2K index, showed that the majority of patients (60.20%) had no or mild disease activity. In contrast, 13.59% (n = 14) exhibited high disease activity (SLEDAI-2K = 11–19), while 0.97% (n = 1) presented with very high activity (SLEDAI-2K ≥ 20) (Table 1).

### 3.2. Annual Direct Medical Cost Distribution

**Figure 1:**
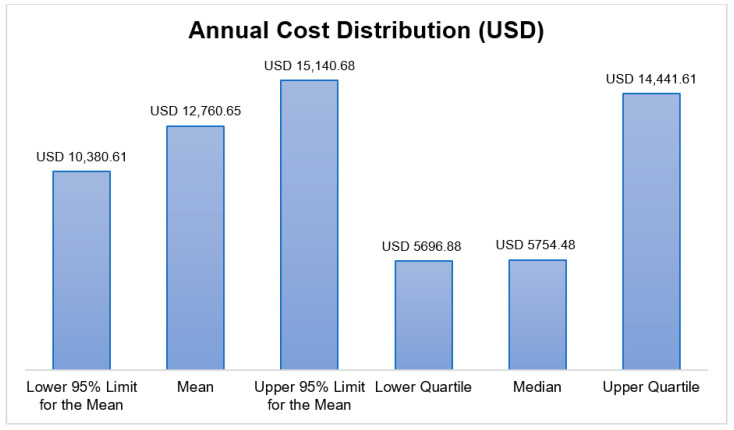
The annual direct medical cost distribution in USD.

**Figure 2:**
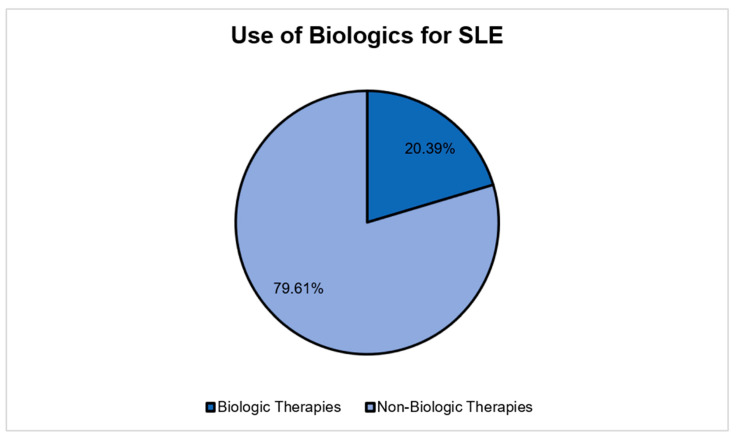
The utilization of biologics among the study sample.

The annual direct medical cost distribution demonstrated a high degree of positive skewness (Figure 1), justifying the use of a Generalized Linear Model (GLM) with a gamma distribution for regression analysis. The utilization of biologic agents, as a key component of high-cost therapy, was captured for the study sample (Figure 2).

The adjusted mean annual direct medical cost per patient, analyzed across various characteristics, is presented in Table 2. Costs remained relatively stable across age groups (ranging from USD 15,973 to 16,614) and gender (USD 16,108 for males vs. USD 16,612 for females).

A strong gradient of increasing cost was observed with rising SLEDAI-2K disease activity:

- No activity (SLEDAI-2K = 0): USD 4047 (95% CI: USD 3648–4491);
- Mild activity (SLEDAI-2K = 1–5): USD 8718 (95% CI: USD 7846–9687);
- High activity (SLEDAI-2K = 11–19): USD 42,996 (95% CI: USD 37,764–48,952);
- Very high activity (SLEDAI-2K ≥ 20): USD 47,793 (95% CI: USD 32,736–69,774).

**Figure 3:**
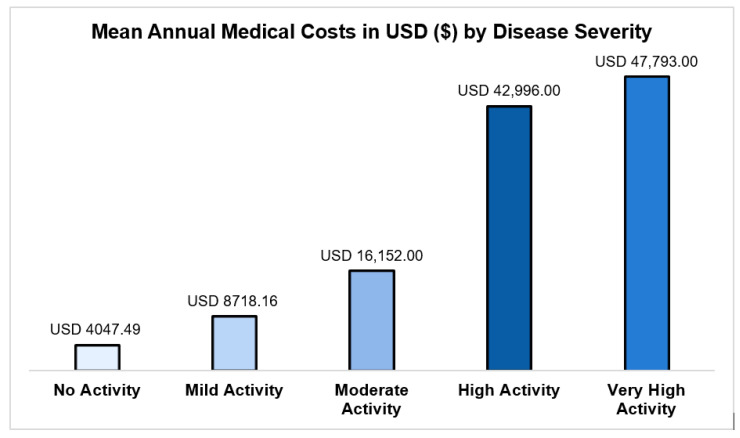
Annual medical costs for SLE by disease severity based on SLEDAI-2K scores.

The adjusted mean annual cost for patients with very high activity was approximately 11.8 times higher than for those with no disease activity (Figure 3). Furthermore, patients receiving biologics incurred significantly higher adjusted mean annual costs (USD 23,041, 95% CI: USD 20,049–26,480) than those not using biologics (USD 11,613, 95% CI: USD 10,352–13,029) (Table 2).

### 3.3. Predictors of Overall Healthcare Costs

The GLM was used to assess the independent contributions of various factors to overall healthcare costs (Table 3). The analysis revealed that age, illness duration, and gender were not statistically significant predictors of healthcare expenses (*p* > 0.05). Conversely, disease activity (estimated coefficient: 0.7397) and the use of biologics (estimated coefficient: 0.7080) were identified as highly significant and independent predictors of increased annual direct medical costs (*p* < 0.0001). These findings indicate that greater disease severity and the need for costly biologic treatments are the primary drivers of healthcare expenditures in this cohort of patients with SLE.

## 4. Discussion

The financial impact of SLE on Saudi Arabia’s public healthcare system is significant, primarily due to acute disease flares and the high costs associated with advanced biologic therapies. This study provides a comprehensive analysis of the variability in direct medical expenditures for SLE management across the country. Notably, annual direct medical costs increase sharply with disease severity. For instance, the average annual expense for managing SLE without disease activity was estimated to be USD 4047, while costs can surge to USD 47,793 for more severe cases, an increase of 11.8 times. This underscores that disease severity represents the most substantial short-term economic burden. These findings align with previously published studies that estimated the cost of illness from the payers’ perspective in different countries [13,21,22,23,24]. In the United States, the mean annual cost using administrative commercial claims data of 2227 adult patients was USD 23,674. The severity of SLE was significantly associated with higher annual medical costs as the mean annual medical cost for patients with mild SLE was USD 12,373 compared to USD 22,559 and 39,261 for those with moderate and severe SLE, respectively [21]. Similarly, in another study that estimated the annual medical cost associated with SLE management in the United States using administrative data and EMRs for patients with private insurance, the mean annual costs of SLE patients were USD 52,951, 28,936, and 21,052 for patients with severe, moderate, and mild SLE, respectively [25]. Additionally, the complications of SLE, such as lupus nephritis, interstitial lung diseases, and avascular necrosis, which are captured in the SLEDAI-2K score [15], were associated with higher annual medical costs among study samples in France, Korea, and the UAE [13,22,26]. Moreover, the findings of this study confirm a strong relationship between SLEDAI-2K score and total annual medical costs, with acute disease flares resulting in the most significant financial strain: a nearly 12-fold increase in costs compared with quiescent disease [15]. Therefore, the SLEDAI-2K index should be utilized as a core quality and cost-control metric.

The use of biologics was associated with more severe forms of SLE and was found to be a significant driver of direct medical costs in multiple studies including a multi-center study that include SLE patients in China and another single-center cost-consequence study that examined the use of belimumab in Saudi Arabia [27,28]. While the use of biologics increases the adjusted mean annual cost for that patient group to USD 23,041, this expenditure is justified as a cost-avoidance measure. Timely and effective utilization of high-cost therapies is essential for refractory patients to achieve and maintain low disease activity (LDA). By achieving LDA, the health system avoids the greater cost burden of severe flares (USD 47,793) and the eventual resource utilization associated with irreversible organ failure. Policy should ensure that access criteria align with a “treat-to-target” strategy, prioritizing effectiveness over short-sighted cost restrictions.

The study provides a reliable baseline mean direct cost of USD 12,761. However, this figure is likely an underestimation of the actual national direct medical cost given the exclusion of patients with expensive SLE-related comorbidities (CKD and CVD). Furthermore, the total economic burden on Saudi society, including productivity losses (a dominant cost factor that has been observed regionally, such as the USD 3.1 billion USD estimated in the UAE), remains unquantified. The national projection, which estimates potential annual direct medical costs ranging from USD 83.8 million (assuming a prevalence rate of 19 patients per 100,000 people) to USD 454.4 million (assuming a prevalence rate of 103 patients per 100,000 people) [4,5], positions SLE as a high-cost condition warranting strategic resource allocation.

### 4.1. Policy Implications

The Ministry of Health (MoH) should designate SLE as a Priority Chronic Disease (PCD) to secure sustained funding. This designation should encompass budget allocations for SLE management, specialized medications, and supportive services such as dialysis for patients with end-stage renal disease. Moreover, national healthcare planning must account for the burden of SLE by ensuring the availability of specialized rheumatology units and essential services, including nephrology and cardiology. Furthermore, policymakers should establish a National SLE Patient Registry spanning both the public and private sectors to generate reliable epidemiological data encompassing clinical outcomes and socioeconomic factors, thereby facilitating the assessment of productivity losses. Funding policies must prioritize health economics research to evaluate the societal cost of this illness. To prevent complications, National Clinical Practice Guidelines (CPGs) should promote early, aggressive treatment strategies to reduce end-organ damage. Additionally, support should be directed toward multidisciplinary Lupus Clinics located in tertiary hospitals, as these have demonstrated effectiveness in decreasing hospitalization rates [29]. Drug procurement policies must guarantee timely access to specialized treatments, guided by regular cost-effectiveness analyses.

### 4.2. Study Limitations

This study represents the first attempt to estimate the annual direct medical costs associated with the management of SLE in Saudi Arabia. However, several limitations must be acknowledged. First, given the single-center design, the generalizability of the findings is limited. Additionally, approximately 60% of the patients exhibited mild or no disease activity, and those with moderate to severe SLE did not require hospitalization or emergency department visits. This could lead to an underestimation of the actual direct medical costs related to SLE management. Furthermore, the analysis was conducted from the perspective of public healthcare payers, who comprise about 70% of the healthcare sector in Saudi Arabia [30]. Additionally, the predominance of nationals and females among the patients hindered the analysis of how gender and ethnicity impacted outcomes and costs. The viewpoint of private healthcare, which represents roughly one-third of healthcare services in the country, was not included in this analysis. As a result, the estimated annual medical costs for SLE management may be very conservative. Furthermore, the study did not account for indirect costs, such as productivity losses, which are believed to be significant. This limitation arises from the study’s focus on the public healthcare payer perspective. Additionally, estimating these indirect costs would require more resources to assess the societal burden of SLE, which is beyond the scope of this investigation.

## 5. Conclusions

The total annual direct medical costs associated with SLE management represent a significant and variable financial burden. In a high regional prevalence scenario, SLE-related expenses could reach approximately USD 454.4 million annually. Given that the Saudi government allocated USD 50.4 billion to healthcare and social development in 2023, this upper estimate for a relatively rare autoimmune disease accounts for nearly 0.90% of the total health budget [31]. Such an expenditure underscores the need for SLE to be recognized as a condition of high economic relevance, necessitating proactive resource planning and its inclusion in the development of the national health strategy under the Vision 2030 initiatives. To comprehensively assess the full economic burden of SLE in Saudi Arabia, future research should transition from single-center studies to national, multicenter epidemiological cohorts. It is essential that these studies adopt a holistic societal perspective to capture productivity losses and involve patients representing the entire spectrum of SLE sequelae, including those with end-stage renal disease (ESRD) and severe cardiovascular comorbidities.

## Figures and Tables

**Table 1:** Patients’ baseline characteristics (n = 103).

Characteristic	Frequency (%)
Age groups	
20–39 years	47 (45.63)
40–59 years	51 (49.51)
≥60 years	5 (4.85)
Gender	
Male	10 (9.71)
Female	93 (90.29)
Nationality	
Saudi	100 (97.09)
Non-Saudi	3 (2.91)
Smoking status	
Smoker	13 (12.62)
Non-smoker	90 (87.38)
Marital status	
Single	41 (39.81)
Married	55 (53.40)
Disease duration	
<5 years	4 (3.88)
5–9 years	28 (27.18)
≥10 years	71 (68.93)
Disease complications	
Proteinuria	21 (20.39)
Hemolytic anemia	6 (5.83)
Leukopenia	21 (20.39)
Arthritis	45 (43.69)
Serositis	6 (5.83)
Lupus nephritis	45 (43.69)
Thrombocytopenia	13 (12.62)
Alopecia	23 (22.33)
Neurological symptoms	9 (8.74)
Chronic cutaneous lupus	21 (20.39)
Baseline systemic lupus erythematosus disease activity index (SLEDAI-2K)	
No activity (SLEDAI = 0)	21 (20.39)
Mild activity (SLEDAI = 1 to 5)	41 (39.81)
Moderate activity (SLEDAI = 6 to 10)	26 (25.24)
High activity (SLEDAI = 11 to 19)	14 (13.59)
Very high activity (SLEDAI ≥ 20)	1 (0.97)

SLEDAI-2K = Systemic Lupus Erythematosus Disease Activity Index 2000.

**Table 2:** The adjusted mean annual direct medical costs across different characteristics.

Characteristic	Cost (USD)	95% Confidence Interval	
		Lower	Upper
Age			
20–39 years	16,614	14,873	18,560
40–59 years	15,973	14,214	17,949
≥60 years	16,494	13,668	19,904
Gender			
Male	16,108	13,859	18,722
Female	16,612	14,871	18,557
Disease Activity			
No activity	4047.49	3647.82	4490.96
Mild	8718.16	7846.39	9686.80
Moderate	16,152	14,553	17,928
High	42,996	37,764	48,952
Very high	47,793	32,736	69,774
Duration of Illness			
<5 years	16,771	13,748	20,458
5–9 years	16,537	14,548	18,798
≥10 years	15,782	14,064	17,711
Use of Biologics			
No	11,613	10,352	13,029
Yes	23,041	20,049	26,480

**Table 3:** Generalized linear regression model with gamma distribution for the relationship between overall healthcare costs and different variables.

Variable	Estimate	95% Confidence Limits		*p*-Value
		Lower	Upper	
Age	−0.0028	−0.0060	0.0004	0.091
Duration of illness	−0.0018	−0.0076	0.0039	0.532
Gender (male versus female)	−0.0102	−0.1351	0.1147	0.873
Disease activity	0.7397	0.7024	0.7771	<0.001 *
Use of biologics	0.7080	0.6146	0.8013	<0.001 *

* *p*-value < 0.05.

## Data Availability

The original contributions presented in this study are included in the article. Further inquiries can be directed to the corresponding author.
